# Human Decidual Stromal Cells in Early Pregnancy Induce Functional Re-Programming of Monocyte-Derived Dendritic Cells *via* Crosstalk Between G-CSF and IL-1β

**DOI:** 10.3389/fimmu.2020.574270

**Published:** 2020-10-27

**Authors:** Qianqian Shao, Xin Liu, Yufei Huang, Xi Chen, Huayang Wang

**Affiliations:** ^1^ Laboratory of Basic Medical Sciences, Qilu Hospital of Shandong University, Jinan, China; ^2^ Department of Ultrasound, Qilu Hospital of Shandong University, Jinan, China; ^3^ Department of Obstetrics and Gynecology, Qilu Hospital of Shandong University, Jinan, China; ^4^ Department of Clinical Laboratory, The Second Hospital of Shandong University, Jinan, China; ^5^ Department of Clinical Laboratory, Qilu Hospital of Shandong University, Jinan, China

**Keywords:** ****decidual stromal cells, dendritic cells, G-CSF, IL-1β, spontaneous abortions

## Abstract

Accumulation of dendritic cells (DCs) is a special characteristic of the decidual microenvironment. Decidua-infiltrated DCs show unique phenotypes and functions that promote the establishment of fetal-maternal tolerance. However, the regulatory mechanisms yet to be fully investigated. Decidual stromal cells (DSCs) are the major cellular component of decidua tissue. The interactions between DSCs and decidua-infiltrated immunocytes dictate immune tolerance in early pregnancy. Therefore, in the present study, we explore the effect of early pregnancy DSCs on monocyte-derived DCs and the relevant mechanisms. DSC-conditioned DCs showed altered phenotypes, secretion profiles and Th2 priming potential. G-CSF concentration was significantly up-regulated in the co-culture supernatant between DSCs and DCs. Supplementation of G-CSF neutralizing antibody partly reversed the reprogramming of DCs mediated by DSCs. Furthermore, G-CSF production was promoted by IL-1β, which was mainly produced by DCs and significantly up-regulated after their cultivation with DSCs. Interestingly, the effects of DSC on IL-1β production of DCs occurred in their immature stage but not their mature stage. Lastly, no significant difference of G-CSF was found in DSCs from healthy early pregnancy women and spontaneous abortions (SA) patients. However, DSCs from SA patients secreted less G-CSF in response to exogenous rhIL-1β or DC cultivation. In conclusion, our study bolster the understanding of the decidual immunomodulatory microenvironment during early pregnancy, and brings new insight into the potential clinical value of G-CSF in pregnancy disorders.

## Introduction

Human pregnancy is a complex process containing interactions between fetal and maternal derived components. Successful embryo implantation depends on significant morphologic and functional changes of the maternal endometrium, a process named decidualization (1). The unique decidual microenvironment not only provides nutritional and endocrine support for embryo implantation and growth, but also plays important immunological roles in the establishment of fetal-maternal tolerance. Fetal-maternal tolerance is an essential adaptation, involving multiple types of decidua-infiltrated immunocytes to facilitate fetus development.

Accumulation of dendritic cells (DCs) is a special characteristic of the decidual microenvironment. Compared to their scarcity in peripheral blood (around 1%) (2), DCs represent approximate 5–10% of all hematopoietic cells in decidua tissue (3, 4). The majority of decidua-infiltrated DCs show myeloid origin with an immature phenotype. More importantly, they exhibit tolerogenic potential through various mechanisms, such as inducing the expansion of regulatory T cells, limiting the proliferation of effector T cells, and driving a Th2-bias response. Tolerogenic DCs play important roles in successful pregnancies in both mice and humans. In mice, depletion of uterine DCs leads to implantation failure and embryo resorption (5). Conversely, inoculation of female CBA/J mice with syngeneic DCs reduced miscarriage rate in the abortion-prone CBA/J × DBA/2J mating model (6). In humans, fewer immature myeloid DCs have been found in the decidua tissues of spontaneous abortion patients compared to normal decidua (7). The researchers speculated that DCs from the spontaneous abortion decidua matured, emigrated to local lymph nodes, and initiated abortifacient Th1 responses. These studies suggest that decidual DCs undergo subtle regulations for their functions and that dysregulation might break the maternal-fetal immune tolerance, leading to a negative pregnancy outcome. However, the regulatory mechanisms yet to be fully investigated.

Decidual stromal cells (DSCs) are the major cellular component of decidua tissue. Numerous studies have reported that the interactions between DSCs and decidua-infiltrated immunocytes dictate immune tolerance in early pregnancy (8). One especially interesting cytokine is granulocyte colony-stimulating factor (G-CSF), which is mainly produced by first trimester decidua cells (9). G-CSF has long been applied as reproductive medicine in the *in vitro* fertilization (IVF) process (10, 11) to stimulate oocyte maturation (12), prevent implantation failures (13, 14), or improve the uterine receptivity in patients with recurrent spontaneous abortion (RSA) (14). There is also evidence that G-CSF may participate in the formation of the tolerogenic microenvironment in decidua by educating locally infiltrated immunocytes. A preliminary study showed that G-CSF abolished IFN-γ production and the cytotoxicity of uterine natural killer (uNK) cells *in vitro* (15). Other researchers further demonstrated its immunomodulatory roles, pointing out that an absence of activated killer-cell immunglobulin-like receptor (KIR)—the functional surface receptor of NKs—was a hallmark of G-CSF application in RSA treatment (16, 17). Studies about G-CSF effects on other types of immunocytes in the pregnancy interface are quite limited. However, when considering the well-recognized G-CSF-mediated induction of tolerogenic DCs in immune-mediated diseases—such as infection, graft-vs-host disease, multiple sclerosis, lupus nephritis and inflammatory bowel disease (18, 19)—it is reasonable to speculate that G-CSF may regulate the phenotypes and functions of decidua-infiltrated DCs.

Based on the above observations, we hypothesize that DSCs regulate the phenotypes and functions of DCs. We investigated this hypothesis by establishing an *in vitro* co-culture system between freshly isolated monocyte-derived DCs and early pregnancy DSCs. We found that DSCs induced functional reprogramming of monocyte-derived DCs, including altered surface marker expression, secretion profile and Th2-driven potential, through a G-CSF-dependent way. Furthermore, DC-derived IL-1β further stimulated G-CSF production. Finally, we analyzed G-CSF production of DSCs from healthy pregnancy women and patients with spontaneous abortions (SA), and found no significant difference of the basal G-CSF secretion level between two groups. However, DSCs from patients with SA did exhibit compromised G-CSF production in response to exogenous rhIL-1β or monocyte-derived DCs by 45.7 and 31.5%, respectively. Our results bolster the understanding of the interactions occurring in the decidual immunomodulatory microenvironment during early pregnancy.

## Materials and Methods

### Collection of Human Samples

This study was approved by the Human Research Ethics Committee of Qilu Hospital of Shandong University (Shandong, China). All subjects signed consent for sample collection and subsequent analysis. First trimester decidua tissues were collected from healthy pregnant women who underwent artificial abortions for non-medical reasons (n = 10, age 28.2 ± 2.8 years; gestational days at sampling 49.8 ± 10.2 days) and spontaneous abortion patients (n = 7, age 27.7 ± 3.9 years; gestational days at sampling 54.7 ± 6.4 days). All the normal pregnancies and miscarriages were confirmed by ultrasound and blood test. All women were non-smokers, not on medication, and had a history of regular menstrual cycles. Chromosomal abnormalities were excluded from the study through analysis of chromosomal karyotype. The decidua tissues were immediately collected into ice-cold sterile RPMI-1640 (HyClone, USA) and transported to the laboratory within 30 min after surgery. Peripheral blood samples were donated from healthy young women volunteers and collected by K2EDTA-containing BD vacutainer (United Kingdom).

### Isolation and Culture of Human DSCs

First trimester human DSCs were isolated and cultured as has been previous described (20). In short, collected decidua tissues were washed in calcium/magnesium-free Hanks balanced salt solution (HBSS) three times and then minced into small pieces. The minced pieces were further digested by 0.1% collagenase type I (Sigma-Aldrich, USA) and 0.25% trypsin (Invitrogen, USA) for 30 min and then filtrated through a sieve (125 μm). The filtrated substances were centrifuged at 120×g for 10 minutes. The supernatant was discarded, and the cell pellet was resuspended by RPMI-1640 with 10% fetal bovine serum (FBS, HyClone, USA) and seeded in culture flasks. After 1 hour the macrophages and granulocytes that adhered to the flasks were discarded and the supernatant was cultured overnight. On the second day, the blood cells in the supernatant were discarded and replaced with new complete RPMI-1640 medium. After 3–4 passages decidua cells were confirmed as vimentin positive and cytokeratin negative and the purity was over 98%.

### Generation of Human Monocyte-Derived DCs

Human peripheral blood mononuclear cells (PBMCs) were obtained by centrifugation with Ficoll-Paque Plus (Sigma-Aldrich). CD14+ monocytes were isolated from PBMCs by positive selection using anti-CD14–conjugated magnetic microbeads (MiltenyiBiotech, Germany) according to the manufacturer’s instructions. The purity was checked by flow cytometry and was above 95%. To generate monocyte-derived immature DCs (imDCs), monocytes were cultured at 1 × 10^6^/mL in complete RPMI-1640 medium containing 1,000 U/mL GM-CSF and 500 U/mL IL-4 (R&D Systems, USA) at 37°C, 5% humidiﬁed CO_2_, for 5 days. To induce maturation (mDCs), imDCs were treated with 100 ng/mL LPS (Sigma-Aldrich) for another 48 h.

### Establishment of Co-Culture System

In co-culture experiments, DSCs (5 × 10^4^ per well) were seeded into a 6-well flat-bottom plate with complete RPMI-1640 medium. CD14^+^ monocytes were seeded into the Transwell chambers (0.4-μm pore size membrane for 6-well plate, Corning) at a ratio of 1:10 (DSCs to monocytes). The chambers were inserted into the DSC-seeded wells for further induction of imDCs or mDCs as above described. In some experiments anti-human G-CSF neutralizing antibody (G-CSF NAb, 4 μg/mL, Abcam, UK) was added into the co-culture system.

### Phenotype Analysis

Surface molecule expression on DCs was measured by FITC-, PE-, APC- and PE-Cy5-labeled monoclonal antibodies: CD1a, CD14, CD80, CD83, CD86, HLA-DR, IL-1R1, and TIMP-3 (all from BD Pharmingen, USA except IL-1R1 from R&D Systems, USA). Isotype controls were performed in parallel. The samples were acquired on a FACSCalibur flow cytometer (BD Biosciences, USA) and analyzed by FlowJo software (FlowJo, USA).

### Cytokines Analysis by ELISA

Cytokines including IL-1β, IL-4, IL-6, IL-8, IL-10, IL-12(p70), IFN-γ, TNF-α, G-CSF, MCP-1, and MIP-1β in the supernatant were quantified using the commercial ELISA kit (all from R&D Systems except G-CSF from Elabscience Biotech, China), according to the manufacturer’s instructions.

### Determination of T-Cell Polarization

CD4^+^T lymphocytes were isolated from PBMC by a naive CD4^+^T cell isolation kit (Miltenyi Biotec) in accordance with the manufacturer’s instructions. Syngeneic mixed lymphocyte reactions were performed based on previous study with some modifications (20). In short, naive CD4^+^ T lymphocytes (5 × 10^5^ per well) isolated from peripheral blood were seeded into 24-well flat bottom plate in complete RPMI 1640 medium containing soluble anti-human CD3 mAb (1 μg/mL, R&D Systems, USA) and rhIL-2 (20 U/mL, R&D Systems, USA) and co-cultured with syngeneic DCs (1 × 10^5^ per well) of different treatment groups for 5 days. The supernatants were harvested and assessed for cytokine production. For intracellular IFN-γ and IL-4 staining, T cells were re-stimulated with 10 ng/mL phorbol myristate acetate (PMA) and 1 μg/mL ionomycin in the presence of 10 μg/mL brefeldin A (all Sigma-Aldrich) for 5–6 h. Cells were then ﬁxed (2% paraformaldehyde), permeabilized (0.5% saponin), and analyzed by ﬂow cytometry using PE-conjugated anti–IL-4 and IFN-γ antibodies (eBioscience, USA).

### Statistical Analysis

Data were achieved from three independent experiments and presented as mean ± SD. Two-tails Student’s t test was conducted for statistical comparisons. All statistical analyses were conducted using GraphPad Prism Version 8.0 (GraphPad Software, USA). P value of less than 0.05 was considered statistically significant.

## Results

### Decidual Stromal Cells Alter the Phenotypic Characteristics of Immature Monocyte-Derived DCs

**Figure 1:**
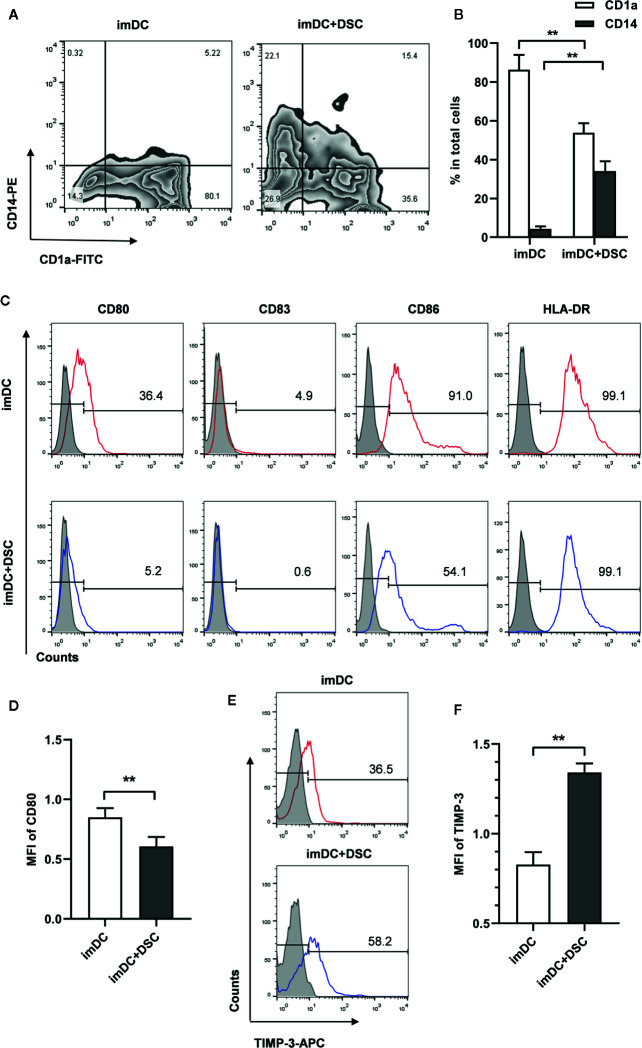
DSCs modulate phenotypes of immature monocyte-derived DCs. **(A)** The expression of CD1a and CD14 on immature monocyte-derived DCs cultivated with DSCs (imDC+DSC) or not was analyzed by flow cytometry and displayed in the form of contour map. A representative result from 5 independent experiments was shown. **(B)** Percentages of CD1a^+^ and CD14^+^ subpopulation in total imDCs. **(C)** The expression of co-stimulatory factors and HLA-DR on imDCs cultivated with DSCs or not was analyzed by flow cytometry. A representative result from 5 independent experiments was shown. Isotype control was shown as gray filled histogram and indicated molecules were shown as open histogram. **(D)** The mean fluorescence intensity (MFI) of CD80 was presented (after Log transformation). **(E)** TIMP-3 expression on imDCs cultivated with DSCs or not was analyzed by flow cytometry, and a representative result from five independent experiments was shown. **(F)** MFI of TIMP-3 was presented (after Log transformation). All experiments were independently conducted for 5 times, and data were presented as mean with SD (**P < 0.01).

In order to investigate whether decidual stromal cells (DSCs) regulate the differentiation from monocytes to DCs and their maturation, CD14^+^ monocytes isolated from PBMC were cultivated with DSCs from healthy early pregnancy women in the presence of GM-CSF/IL-4. Monocytes-derived immature DCs (imDCs) were collected after 5 days cultivation and the expression of surface markers of DCs analyzed by flow cytometry. As **Figures 1A, B** show, a significant change was observed in the expression of CD1a and CD14. Control imDCs were mostly negative for CD14 (4.25 ± 1.33%), and the majority were positive for CD1a expression (86.30 ± 7.65%). Significantly higher percentages of CD1a^-^ and CD14^+^ subpopulations were found in imDCs cultivated with DSCs (55.08 ± 4.64% for CD1a and 34.08 ± 5.04% for CD14).

We next explored the expression of major histocompatibility complex (MHC) class II molecules and costimulatory molecules, such as CD80, CD83, and CD86 at the surface of imDCs. Here we found that a significant decrease of CD80+ percentage in imDCs co-cultured with DSCs (34.36 ± 4.07% vs. 4.44 ± 0.94%). A similar decrease was also found in CD86 expression (89.52 ± 3.07% vs. 54.90 ± 1.97%, **Figure 1C**). A significant decrease of expression intensity of CD80, as determined by mean fluorescence intensity (MFI), was found in DSC-cultivated imDCs, compared with controls (0.85 ± 0.08 vs. 0.61 ± 0.08 after Log transformation, **Figure 1D**).

We further analyzed TIMP-3 expression in DSC-conditioned imDCs, as TIMP-3 has been demonstrated to be involved in the differentiation of DCs by our previous study (20). We found that both the positive percentage (32.74 ± 5.81% vs. 60.54 ± 4.21%) and expression intensity (0.83 ± 0.01 vs. 1.34 ± 0.01 after Log transformation) of TIMP-3 were significantly up-regulated in DSC-conditioned imDCs compared with controls (**Figures 1E, F**).

### DSC-Conditioned Mature DCs Display Altered Cytokine Profiles and Th2 Priming Potential

**Figure 2:**
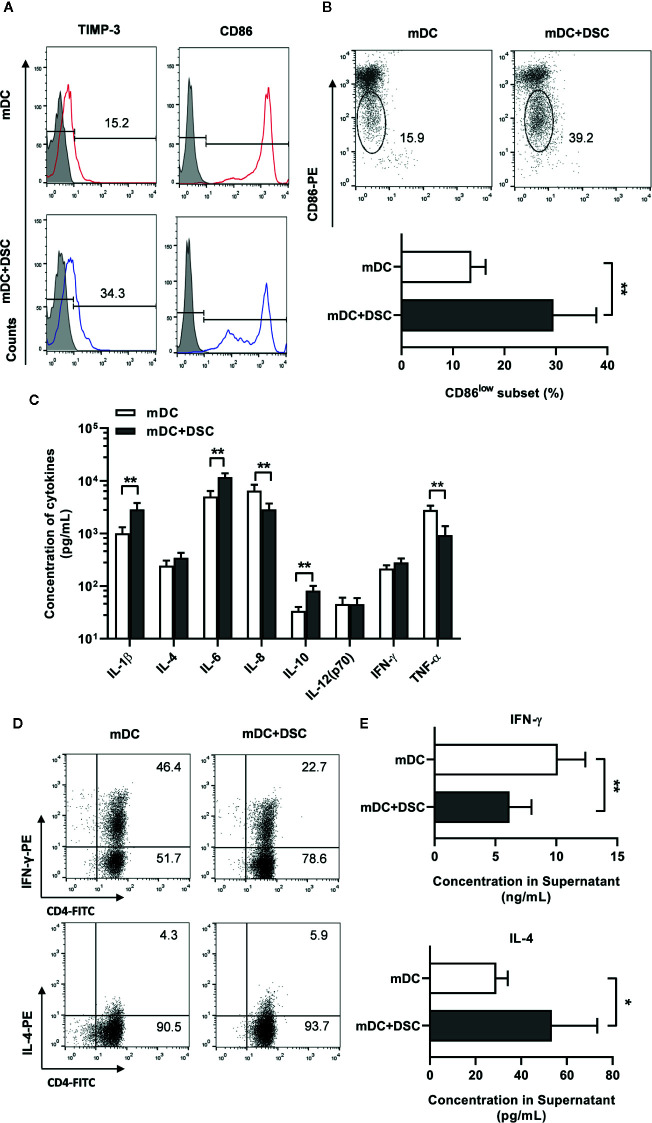
DSCs modulate the phenotype, secretion profile and T cells priming potential of mature DCs. **(A)** Surface expression of CD86 and TIMP-3 on mature DCs cultivated with DSCs (mDC + DSC) or not was analyzed by flow cytometry. A representative result from 5 independent experiments was shown. **(B)** A representative result of CD86^low^ subset of DCs of two separate group (upper) and its percentage (lower) was shown. **(C)** ELISA results of cytokines concentration in conditioned medium of mDCs were shown. **(D)** mDCs and DSC-conditioned DCs were incubated with syngeneic naive CD4^+^T cells for 5 days. Intracellular expression of IFN-γ (upper) and IL-4 (lower) in T cells were stained and analyzed by flow cytometry. A representative result from 5 independent experiments was shown. **(E)** Supernatant of the mixed lymphocyte reaction between DCs and T cells were collected, and concentrations of IFN-γ (upper) and IL-4 (lower) in the supernatant were analyzed by ELISA. All experiments were conducted for 5 times independently, and data were presented as mean with SD (*P < 0.05; **P < 0.01).

We generated mature DCs (mDCs) by stimulating imDCs with LPS (100 ng/mL) for 48 h. First, we analyzed the phenotypes of mDCs and found no significant change in most surface markers between the control and the DSC-conditioned groups (Data not shown). Notably, we found a significant increase of positive percentage (17.58 ± 2.63% vs. 33.44 ± 5.01%) of TIMP-3 in DSC-conditioned mDCs (**Figure 2A**). Moreover, although CD86 was positive on almost all mDCs (**Figure 2A**), we observed an increase of proportion of CD86^low^ subset in DSC-conditioned mDCs (13.54 ± 2.80% vs. 29.52 ± 8.31%, **Figure 2B**), which indicated a less mature status.

We went on to explore whether cultivation with DSCs would modulate the secretion profile of DCs. mDCs were separated from DSCs after period of cultivation, washed and cultured alone for another 24 h, and then the conditioned medium (CM) was collected. We found significant up-regulation of IL-1β, IL-6, and IL-10 and down-regulation of IL-8 and TNF-α in CM of DSC-conditioned mDCs (**Figure 2C**).

We further tested the T cells priming potential of DCs by stimulating naive T cells with mDCs from the control or the DSC-conditioned groups. Intracellular staining assays showed that T cells primed by control mDCs exhibited a higher percentage of IFN-γ-producing groups than IL-4-producing groups, indicating a Th1 priming potential for control mDCs (**Figure 2D**). In contrast, T cells primed by DSC-conditioned mDCs exhibited a Th2 profile, supported by a decrease of IFN-γ-producing percentage and an increase of IL-4-producing percentage (**Figure 2D**). The Th2 priming potential was also revealed, as IFN-γ was significantly decreased (10.12 ± 2.27 vs. 6.15 ± 1.08 ng/mL) and IL-4 was significantly increased (29.06 ± 5.02 vs. 53.42 ± 19.90 pg/mL) in the co-cultured supernatant between naive T cells and DSC-conditioned mDCs (**Figure 2E**).

### G-CSF Is Partially Involved in DSC-Induced Phenotype and Function Modulation of Monocyte-Derived DCs

**Figure 3:**
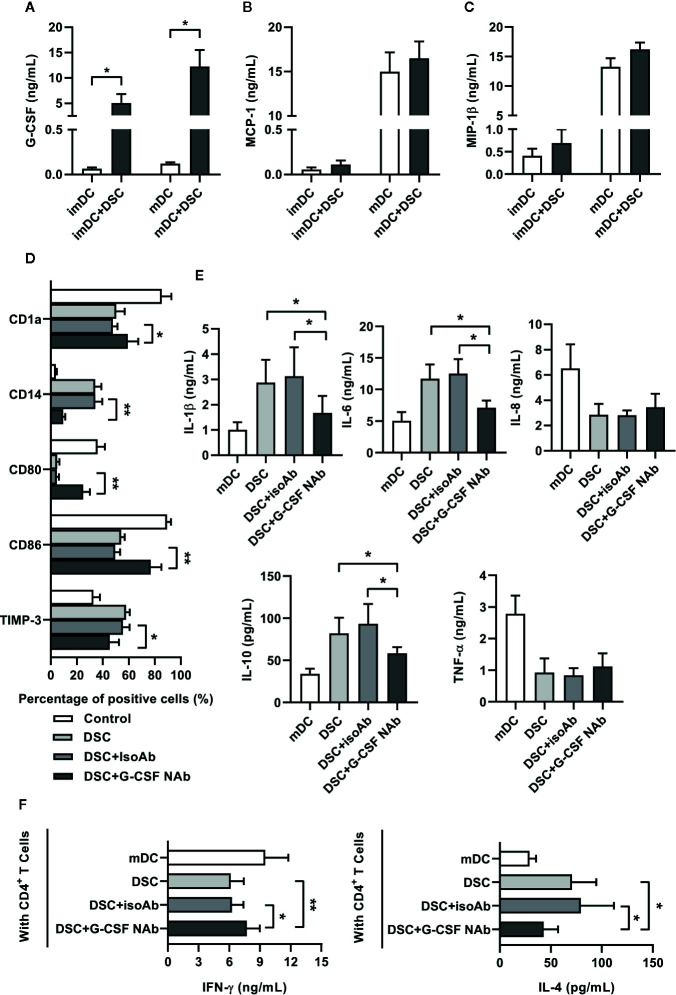
G-CSF was involved in DSC-mediated functional reprogramming of DCs. **(A–C)** Co-culture supernatant between DSCs and DCs were collected at day 5 or day 7 as indicated, and concentrations of G-CSF **(A)**, MCP-1 **(B)** and MIP-1β **(C)** were analyzed by ELISA. **(D)** The phenotypes of imDCs, cultivated with DSCs in the presence of G-CSF neutralizing antibody (NAb) or not were analyzed by flow cytometry. Percentages of CD1a, CD14, CD80, CD86 and TIMP-3-positive subpopulations in total imDCs were shown. IsoAb indicated isotype control. **(E)** ELISA results showed concentrations of cytokines in the conditioned medium of mDCs, cultivated with DSCs in the presence of G-CSF neutralizing antibody (NAb) or not. **(F)** mDCs, cultivated with DSCs in the presence of G-CSF neutralizing antibody (NAb) or not, were incubated with syngeneic naive CD4^+^T cells for 5 days. Concentrations of IFN-γ and IL-4 in the supernatant of the mixed lymphocyte reaction between DCs and T cells were shown. All experiments were conducted for five times independently, and data were presented as mean with SD (*P < 0.05; **P < 0.01).

We speculated that soluble factors might be involved in the modulation of DSCs on conditioning monocyte-derived DCs. Therefore, we analyzed the concentrations of some factors in the co-cultured supernatant—which have been proven to be involved in the differentiation and maturation of DCs—including G-CSF, MCP-1, and MIP-1β. Of the three factors, only G-CSF showed a substantial up-regulation (0.06 ± 0.01 ng/mL in imDC vs. 5.03 ± 1.78 ng/mL in imDC+DSC and 0.12 ± 0.01 ng/mL in mDC vs. 12.27 ± 3.25 ng/mL in mDC+DSC) in the co-cultured supernatant compared to CM of DCs (**Figures 3A–C**).

To test the roles of G-CSF in the conditioning of monocyte-derived DCs by DSCs, we added anti-G-CSF neutralizing antibody (NAb) into the co-cultured system. As **Figure 3D** shows, the presence of G-CSF NAb recovered the expression of surface markers of imDCs. The expression of CD14, CD86, and TIMP-3 were restored in the presence of G-CSF NAb, whereas the expression of CD1a and CD80 were also significantly elevated. Moreover, we also found that G-CSF NAb greatly inhibited IL-1β, IL-6, and IL-10 expression of mDCs stimulated by DSCs. However, G-CSF NAb showed no significant effect on IL-8 and TNF-α (**Figure 3E**). Finally, we investigated whether the presence of G-CSF NAb in the co-culture system would regulate the subsequent T cells priming potential of DCs. As shown by **Figure 3F**, in the mixed lymphocyte reaction between naive T cells and G-CSF NAb previously treated DCs, the concentrations of IFN-γ and IL-4 were partly restored.

### DC-Derived IL-1β Is Up-Regulated by DSCs During the Monocyte-imDC Phase and Promotes G-CSF Production

**Figure 4:**
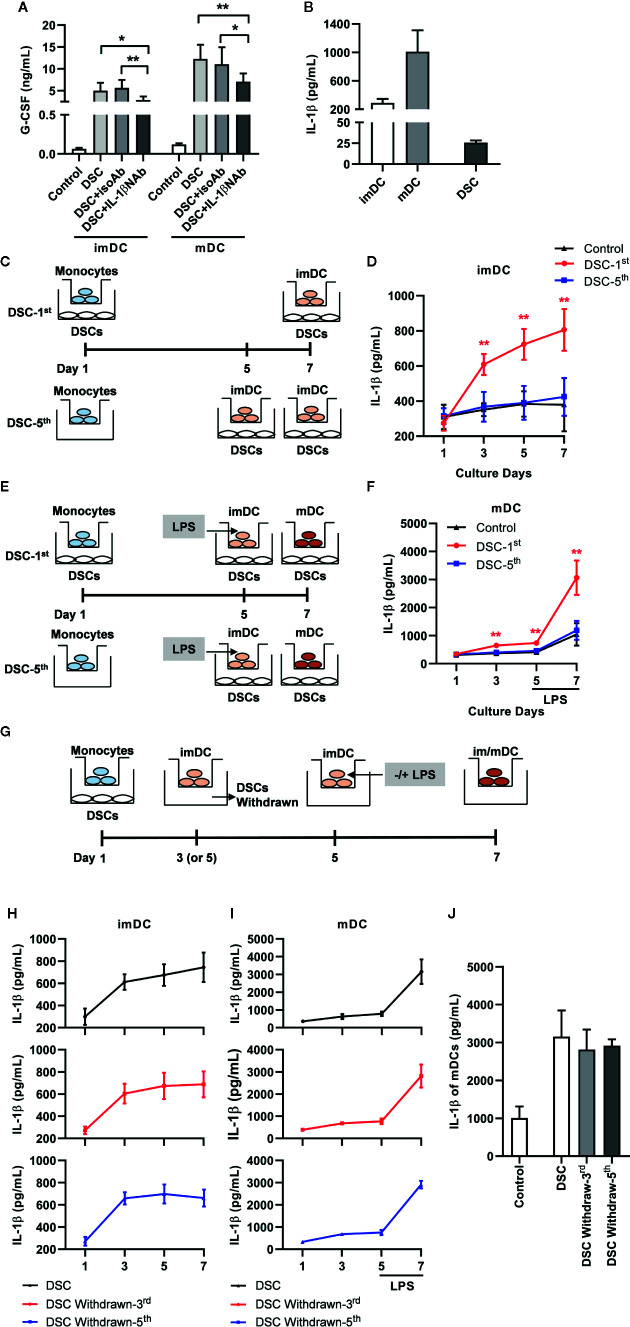
IL-1β produced by DCs, which was up-regulated by G-CSF during immature stage, promoted G-CSF secretion in the co-culture system. **(A)** Concentration of G-CSF in the co-culture supernatant between DSCs and imDCs or mDCs, in the presence of IL-1β NAb or not. **(B)** Concentration of IL-1β in the conditioned medium of imDCs, mDCs and DSCs. **(C–F)** IL-1β concentration in conditioned medium with different cultivation procedure between DCs and DSCs. Diagrammatic illustrations showed different time at which DSCs were added into the co-culture system with imDCs **(C)** and mDCs **(E)**. DSC-1^st^: DSCs were added into the system at 1^st^ day of DCs cultivation. DSC-5^th^: DSCs were added into the system at 5^th^ day of DCs cultivation. imDCs or mDCs were collected at day 7 as indicated. IL-1β concentration in conditioned medium of imDCs **(D)** and mDCs **(F)** were measured by ELISA. The experiments were independently repeated for five times. **(G)** A diagrammatic illustration showed different time at which DSCs were withdrawn from the co-culture system. **(H, I)** IL-1β concentration in conditioned medium of imDCs **(H)** and mDCs **(I)** from different treatment groups, in which DSCs were withdrawn at different time, were measured by ELISA. The upward arrows indicated DSC withdrawn. **(J)** IL-1β concentration in conditioned medium of mDCs at day 7. All experiments were independently conducted for five times. Data were presented as mean with SD (*P < 0.05; **P < 0.01).

In the above results, we noticed that DCs conditioned by DSCs secreted a significantly higher level of IL-1β, which had been proved to stimulate G-CSF production (21). Similarly, we found that the presence of an IL-1β neutralizing antibody significantly inhibited G-CSF production by 41.01% in imDCs and by 42.00% in mDCs in the co-cultured system (**Figure 4A**). We also demonstrated that both imDCs and mDCs produce high levels of IL-1β (288.20 ± 59.25 pg/mL in imDCs and 1008.00 ± 303.10 pg/mL in mDCs), whereas the production of IL-1β by DSCs was scarce (25.74 ± 2.61 pg/mL, **Figure 4B**).

Interestingly, we found that when DSCs were added into the co-culture system at day 5 (**Figures 4C, E**: DSC-5^th^)—that is, the CD14^+^ monocytes had been transformed into imDCs—their promoting effect on IL-1β production for both imDCs and mDCs was greatly compromised (**Figures 4D, F**). On the contrary, when DSCs were added at day 1 and removed from the co-culture system at day 3 or day 5, respectively (**Figure 4G**), the levels of IL-1β secreted by imDCs were no difference to those secreted by DSC-conditioned ones (**Figure 4H**). As for mDCs, DSCs withdraw caused a milder but not significant compromise of IL-1β production in response to LPS at day 7 (**Figures 4I, J**).

### DSCs From Patients With Spontaneous Abortion Produce Lower Levels of G-CSF in Response to Exogenous IL-1β and Conditioning of DCs

**Figure 5:**
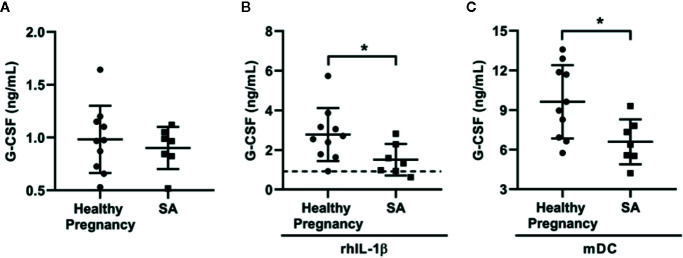
DSCs from SA patients showed low G-CSF production in response to rhIL-1β and DCs condition. DSCs were collected from healthy early pregnant women (n = 10) and patients with spontaneous abortion (SA, n = 7). **(A)** Same amount of DSCs from two groups were cultured for 7 days and the conditioned medium were collected. G-CSF concentration in the conditioned medium was measured by ELISA. **(B)** DSCs from healthy pregnant women and SA patients were cultured for 7 days with the treatment of recombinant human IL-1β (rhIL-1β) for 7 days and G-CSF concentration was measured. Dash line indicated average G-CSF concentration in the conditioned medium of DSCs before rhIL-1β stimulation. **(C)** DSCs from healthy pregnant women and SA patients were co-cultured with mDCs from healthy women for 7 days, and G-CSF concentration was measured. Data were presented as mean with SD (*P < 0.05).

G-CSF administration has been used as a promising treatment option for patients with SA. Therefore, we explored whether the G-CSF secretory capacity of DSCs was compromised in patients with SA. DSCs, collected from patients with SA (n = 7) and healthy pregnant women in their first trimester (n = 10), were cultivated alone with exogenous IL-1β or monocyte-derived DCs for 7 days, respectively. As shown in **Figure 5A**, the basic level of G-CSF secreted by DSCs from patients with SA was no different than healthy pregnant women. However, DSCs from patients with SA secreted significantly less G-CSF in response to exogenous rhIL-1β than those from the healthy controls (1.51 ± 0.79 vs. 2.78 ± 1.33 ng/mL, **Figure 5B**). Moreover, when cultivated with monocyte-derived DCs, the upregulation of G-CSF production by DSCs from patients with SA was further compromised, compared with those from healthy pregnant women (**Figure 5C**, 6.59 ± 1.70 vs. 9.62 ± 2.77 ng/mL).

The lower concentration of G-CSF in supernatant of SA-DSCs under treatment of rhIL-1β was not caused by different cell number, as the proliferation between DSCs from healthy pregnancy and SA patients showed no difference (**Supplemental Figure 1A**), and significant difference of G-CSF production was observed after 1 day of rhIL-1β treatment (0.25 ± 0.13 vs. 0.10 ± 0.05 ng/mL, **Supplemental Figure 1B**). In addition, the expression of IL-1β receptor, IL-1R1, also showed no difference between two types of DSCs (**Supplemental Figure 1C**).

## Discussion

The decidua plays important roles in embryo implantation and maintenance during early pregnancy by forming a unique tolerogenic microenvironment. The maternal-fetal interface contains several types of immunocompetent cells, and their functions are closely regulated by the interactions between each other, or with non-immunocytes. In the present study, we demonstrate that human decidual stromal cells (DSCs) in early pregnancy can modulate the phenotype and functions of monocyte-derived DCs through G-CSF, which promotes the induction of maternal-fetal tolerance. By using indirect *in-vitro* co-culture model, we found that monocyte-derived DCs cultivated with DSCs possess a higher percentage of CD1a^−^ and CD14^+^ subpopulations. DSC-conditioned DCs also express lower levels of maturation markers, CD80, CD86, and higher levels of TIMP-3. Altered cytokine profiles are found in DSC-conditioned DCs associated with Th2-priming potential. Partly stimulated by DC-derived IL-1β, the levels of G-CSF are strikingly elevated in the co-culture system. The upregulation of G-CSF is in turn, involved in the reprogramming of DCs. Finally, we found that DSCs from patients with spontaneous abortion (SA) produce significantly lower levels of G-CSF compared to healthy pregnant women in response to exogenous IL-1β or monocyte-derived DCs.

The phenotypes of decidual DCs is a complex and controversial topic, possibly because they are composed of multiple heterogeneous subpopulations. Nevertheless, most studies agree that decidual DCs exhibit an immature phenotype, with little or no expression of CD1a and reduced expression of CD80 and CD86. In the present study, we find that DSC-conditioned DCs showed immature and tolerogenic features. First, DSC-conditioned DCs display significant amplification of the CD14^+^ and decrease of the CD1a^+^ subpopulations, exhibiting a phenotype similar to intra-decidual antigen presenting cells (APCs) *in situ* (22, 23). Second, DSC-conditioned DCs show a significant decrease in surface expression of co-stimulatory factors (CD80 and CD86) and HLA-DR. As the expression intensity of co-stimulatory factors are greatly enhanced during the maturation of DCs, this decreased expression suggested an immature status. Third, DSC-conditioned DCs secret reduced levels of pro-inflammatory cytokines IL-8 and TNF-α, and enhanced levels of anti-inflammatory cytokine IL-10. Last, DSC-conditioned DCs stimulate T cells to produce less Th1-type cytokine (IFN-γ) but more Th2-type cytokines (IL-4), indicating the Th2 priming potential of DSC-conditioned DCs.

Our previous study found that surface expression of TIMP-3 effectively suppressed CD86 expression and IL-12 production of DCs, leading to a Th2 polarization of naive T cells (20). In the present study, we analyze the expression of TIMP-3 in DSC-conditioned DCs and find that TIMP-3 is significantly upregulated after co-culture with DSCs. Notably in our previous studies, although the upregulation of TIMP-3 occurred during the differentiation from monocytes to imDCs, it only caused expression changes of co-stimulatory factors, MHC molecules, and secretion of cytokines after LPS and TNF-α priming (20). In the present study, the upregulation of TIMP-3 expression is observed in both imDCs and mDCs. These observations indicate that enhanced TIMP-3 expression may regulate the functions of DSC-conditioned DCs during the maturation stage, a possibility that should be examined in future studies. Moreover, the mechanisms of TIMP-3 on the regulation of functions of DSC-conditioned DCs also needs detailed investigation.

The underlying mechanisms by which DSCs modulate the differentiation and functions of DCs are largely unknown. In the present study, we found that G-CSF is strikingly upregulated in a co-culture system of DSCs and monocyte-derived DCs. G-CSF and its receptors are expressed on both the fetal (cytotrophoblasts and syncytiotrophoblasts) and the maternal (DSCs, endometrial glands and epithelium) sides (24). The local production of G-CSF in the fetal-maternal interface modulates the cytotoxicity of uterine nature killer cells (NKs) and reduces their pro-inflammatory cytokines production (15). Our study demonstrates that the presence of a G-CSF neutralizing antibody partly restores the phenotype of DSC-conditioned DC, with decreased CD1a^-^ and CD14^+^ subpopulation percentages, and increased CD80 and CD86 expression. Moreover, G-CSF NAb also recovers the cytokine profiles of DCs. To our knowledge, this is the first report showing that G-CSF regulates the functions of DCs in maternal-fetal interface.

During the analysis of cytokine secretion profiles of DSC-conditioned DCs, we found a significant enhancement of IL-1β regulated by G-CSF. IL-1β is well-recognized as key regulator of the inflammatory response and is closely involved in the reproduction process. Previous studies have shown that repeated injections of an antagonist of IL-1 receptor into pregnant mice prior to implantation causes implantation failure (25, 26). In humans, women with habitual abortion show decreased serum levels of IL-1β (27). Moreover, detectable serum IL-1β levels at the start of *in vitro* fertilization (IVF) cycle is associated with a successful IVF outcome (28). Our results suggest a potential link between the controllable inflammatory process and successful implantation. DCs act as one of the major APCs that trigger immune reaction and regulate the intensity of the inflammatory response. Furthermore, previous studies have shown that endometrial biopsy induces DCs accumulation, elevates pro-inflammatory cytokine secretion and promotes successful implantation in IVF patients (29). Our results may reflect a novel mechanism that facilitates pregnancy outcome as mediated by high G-CSF during early stage of pregnancy.

It should also be noted that IL-1β may regulate G-CSF production. A previous study has shown that upregulation of G-CSF in chorio-decidua during chorioamnionitis is IL-1β dependent (30). In our present study, we also demonstrated this positive feedback interaction by showing that supplementation of IL-1β NAb inhibited G-CSF concentration in the co-culture supernatant. According to previous works, G-CSF and IL-1β may work in concert to establishment a special immune niche that facilitates embryo implantation. For example, one study demonstrated that IL-1β induces chemoattractant CXCL1 (31) and CXCL8 (30) expression in DSCs. As their major receptor, CXCR2, is highly expressed in the circulating monocytes of humans, we speculate that IL-1β overexpression in the first-trimester decidua promotes monocytes recruitment into the pregnancy site. High G-CSF level in decidua will further promote the differentiation of recruited monocyte into DCs with immunoregulatory functions, which facilitate embryo implantation and maintenance.

G-CSF has been applied as innovative therapy for early pregnancy disorders, such as repetitive implantation failures and recurrent spontaneous abortions (RSAs). Some clinical trials have been conducted and show promising results (32, 33). However, researchers argue about the indications for G-CSF treatment, as there is no precise definition of an “implantative G-CSF deficiency syndrome” (14). One possible solution has used KIR typing from uterine NK cells (uNK), with the hypothesis that G-CSF dysfunction leads to the disruption of activating interactions (16, 34). This solution is still questionable however, especially when considering that tissue-infiltrated immunocytes are under the effects of variable factors and G-CSF may not be the primary factor. In the last part of our study, we analyzed G-CSF expression of DSCs from healthy pregnant women and SA patients. It was surprising to find that the basal secretion of G-CSF of DSCs from SA patients were no different with those from healthy pregnancy women. However, DSCs from SA patients produced significantly less G-CSF in response to exogenous IL-1β and DCs stimulation. Our findings suggest that the pregnancy interface of SA patients may not undergo an absolute deficiency of G-CSF but exhibit compromised G-CSF secretion under certain stimulations. The compromise appear after 1 day of IL-1β treatment and no difference of expression of IL-1β receptor, IL-1R1, was found. Therefore whether this compromise comes from aberrant intracellular signaling pathways or imbalanced cytokine profile needs further investigation. Demonstrating the relevant mechanisms would bring strong support for the supplementation of G-CSF as reproductive medicine and may also shed light on the establishment of a more direct and reliable indication criteria.

In conclusion, our results suggest that DSCs regulate the phenotypes and functions of monocyte-derived DCs, and that this effect is mediated by a unique positive feedback interaction between G-CSF and IL-1β. Our study provides new evidence for the regulatory mechanisms behind the establishment of maternal-fetal tolerance at the early stage of pregnancy and brings new insight into the potential clinical value of G-CSF in pregnancy disorders.

## Data Availability Statement

All datasets presented in this study are included in the article/**Supplementary Material**.

## Ethics Statement

The studies involving human participants were reviewed and approved by the Human Research Ethics Committee of Qilu Hospital of Shandong University. The patients/participants provided their written informed consent to participate in this study.

## Author Contributions

QS performed the research and analyzed data. XL, YH, and XC performed the research. QS and HW designed the research. HW further analyzed data and wrote the paper. All authors contributed to the article and approved the submitted version.

## Funding

This study was supported by National Natural Science Foundation of China (grant nos. 81702815 and 81300510) and Key Technology Research and Development Program of Shandong, China (grant nos. 2019GSF108247).

## Conflict of Interest

The authors declare that the research was conducted in the absence of any commercial or financial relationships that could be construed as a potential conflict of interest.

## Supplementary Material

Supplemental Figure 1 DSCs from SA patients showed no difference in proliferation and IL-1R1 expression with healthy pregnant DSCs. **(A)** Cell viability of DSCs from healthy pregnancy (n = 6) and SA patients (n = 6) was analyzed by CCK-8 test. **(B)** G-CSF concentration in supernatant of DSCs from healthy pregnancy (n = 6) and SA patients (n = 6) after 1 day, 3 days, 5 days, and 7 days of rhIL-1β treatment. **(C)** IL-1R1 expression was analyzed in healthy pregnant and SA DSCs by flow cytometry. (Left) Eight representative figures from 12 independent specimens were shown. (Right) Percentage of IL-1R1-positive DSC subset in healthy pregnant women (n = 6) and SA patients (n = 6). Data were presented as mean with SD (**P* < 0.05).
