# Supplementary figures and images for: Human Decidual Stromal Cells in Early Pregnancy Induce Functional Re-Programming of Monocyte-Derived Dendritic Cells via Crosstalk Between G-CSF and IL-1β
Source: Front Immunol. 2020 Oct 27;11:574270. doi: 10.3389/fimmu.2020.574270 (PMC7652738; doi:10.3389/fimmu.2020.574270)

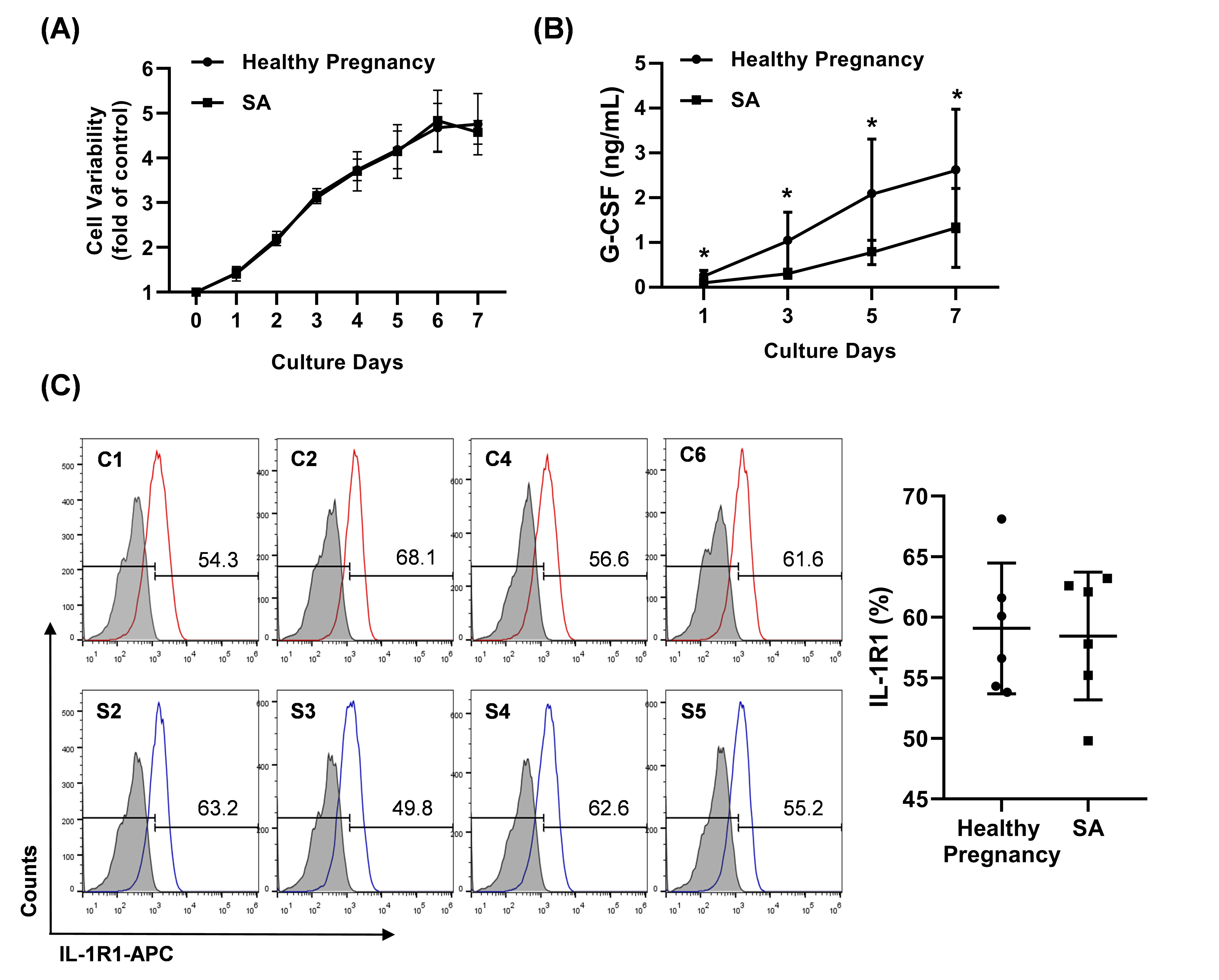

Supplement: Supplemental Figure 1 — DSCs from SA patients showed no difference in proliferation and IL-1R1 expression with healthy pregnant DSCs. (A) Cell viability of DSCs from healthy pregnancy (n = 6) and SA patients (n = 6) was analyzed by CCK-8 test. (B) G-CSF concentration in supernatant of DSCs from healthy pregnancy (n = 6) and SA patients (n = 6) after 1 day, 3 days, 5 days, and 7 days of rhIL-1β treatment. (C) IL-1R1 expression was analyzed in healthy pregnant and SA DSCs by flow cytometry. (Left) Eight representative figures from 12 independent specimens were shown. (Right) Percentage of IL-1R1-positive DSC subset in healthy pregnant women (n = 6) and SA patients (n = 6). Data were presented as mean with SD (*P < 0.05). [file Image_1.tif]
